# Tubulovillous adenoma with high-grade dysplasia of the vulva harboring high tumor mutational burden and cancer-associated mutations: a case report

**DOI:** 10.1186/s13000-022-01268-7

**Published:** 2022-10-28

**Authors:** Hanako Sato, Kosuke Murakami, Tomoyuki Otani, Noriomi Matsumura

**Affiliations:** 1 Department of Obstetrics and Gynecology, Kindai University Faculty of Medicine, 377-2 Ohnohigashi, Osakasayama, Osaka Japan; 2 Department of Pathology, Yachiyo Hospital, Anjo, Aichi Japan

**Keywords:** Adenocarcinoma of intestinal-type, Tumor mutational burden, Tubulovillous adenoma, Vagina, Vulva, Vulvar cancer

## Abstract

**Background:**

Vulvar cancer is a rare disease, accounting for approximately 5% of gynecological malignancies. Primary adenocarcinoma of intestinal-type of the vulva or its precancerous lesion is extremely rare, and details regarding its origin, evolution and related genetic mutations are unknown. Treatment options for this cancer have not been defined.

**Case presentation:**

A 63-year-old Japanese woman came to the hospital because she was aware of a vulvar mass. There was a 1 cm mass on the dorsal side of the vulva, just outside the remains of the hymen. Biopsy revealed suspected adenocarcinoma, and wide local excision was performed. From histopathology and immunohistochemistry, the specimen was diagnosed as tubulovillous adenoma with high-grade dysplasia of the vulva. No other primary lesions were found, and the vulva was considered the primary site. A gene panel test (FoundationOneCDx assay) showed a high tumor mutational burden and mutations in *TP53*, *KEL*, *RB1*, *RNF43*, *PTEN*, *GNAS*, and *PIK3CA*.

**Conclusions:**

The current case of tubulovillous adenoma with high-grade dysplasia of the vulva had a variety of cancer-associated mutations, despite being a precancerous lesion. In cases of intestinal-type neoplasms of the vulva, it may be helpful to check tumor mutational burden and gene mutations for treatment selection.

## Background

Vulvar cancer accounts for only approximately 3–4% of gynecologic malignancies [1]. Approximately 80% of vulvar cancers are squamous cell carcinoma, followed by basal cell carcinoma, Paget's disease, and melanoma; adenocarcinoma is very rare [1]. Adenocarcinoma of intestinal-type of the vulva was first reported in 1964 [2] and is diagnosed based on characteristic pathologic findings and immunohistochemistry (IHC).

Several theories have been reported regarding the origin of intestinal-type neoplasms of the vulva: the urethra, lower vaginal area, and rectum are derived from the cloaca, so the lower vaginal area contains remnants of bowel tissue [3]; ectopic bowel epithelium or intestinal metaplasia within the tissue derived from Müllerian duct [4]; and Bartholin’s glands in the vulva [5]. The precise origin and the evolution of intestinal-type neoplasms of the vulva remain unknown and there is no established treatment. Additionally, the genetic mutation patterns of intestinal-type neoplasms of the vulva have not been investigated.

Here, we report a case of tubulovillous adenoma with high-grade dysplasia of the vulva, as a precancerous lesion of intestinal-type neoplasm of the vulva, with multiple cancer-associated mutations and high tumor mutational burden (TMB).

## Case presentation

**Fig. 1:**
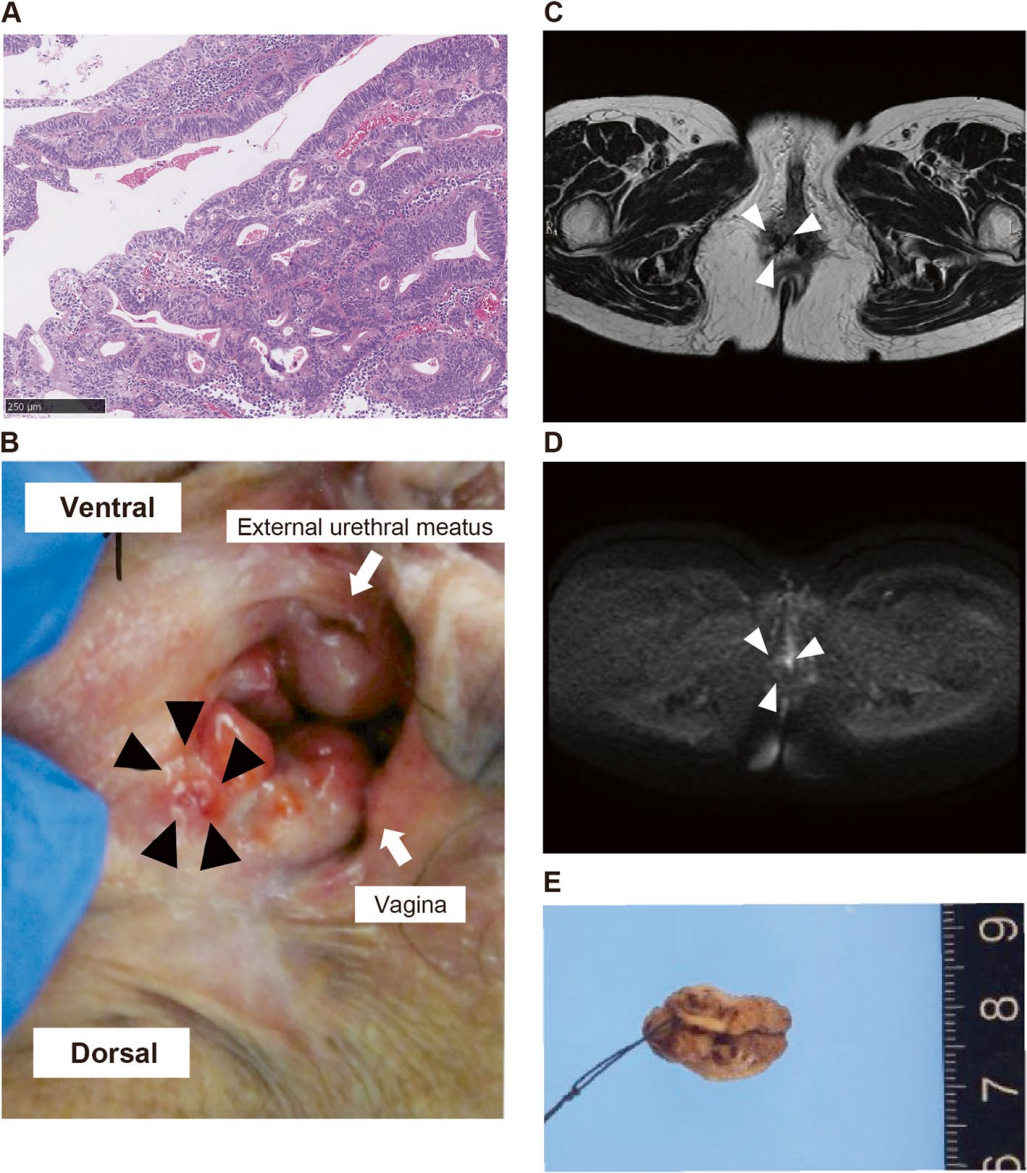
MRI and visual findings (after biopsy). **A** Hematoxylin–eosin staining of the biopsy specimen. Magnification: 100 × , scale bar: 250 µm. **B** Visual findings. There was a red lesion at the vaginal entry at 7 o'clock (black arrowhead). **C** MRI T2-weighted image (axial). The lesion showed a high signal (white arrowhead). **D** MRI diffusion-weighted image (axial). The lesion showed restricted diffusion (white arrowhead). **E** Surgical specimen. The thread indicates the ventral direction

A 63-year-old Japanese woman, gravida 4, para 3, was aware of a mass in the vulva and visited the clinic. She has no medical or family history and no smoking history. There was a 1 cm mass on the vulva that had self-destructed. Biopsy revealed a dysplastic glandular neoplasm (Fig. 1A), and the histopathological diagnosis was adenocarcinoma. The pathological findings were not typical for vulvar primary cancer, and metastatic carcinoma of gastrointestinal origin was suspected. The patient was referred to our hospital.

Gross findings at the time of the visit to our hospital were redness with ulceration of approximately 5 mm just outside the 7 o'clock remains of the hymen, and the mass had disappeared (Fig. 1B). On magnetic resonance imaging, no obvious mass was detected on both T1-weighted and T2-weighted images, but on the diffusion-weighted image, there was a high-signal area of approximately 5 mm that was presumed to be a tumor lesion (Fig. 1C, D). Contrast-enhanced computed tomography and positron emission tomography showed no enlarged lymph nodes or distant metastases. Tumor markers were as follows: CEA, 1.8 ng/mL; CA125, 12 U/mL; and CA19-9, 40 U/mL. Only CA19-9 was slightly elevated. Upper gastrointestinal endoscopy revealed erosions in the gastric angulus, from which one biopsy was performed, but no malignant findings were observed. Lower gastrointestinal endoscopy revealed no abnormal findings. Wide local excision of the vulvar tumor was performed under spinal anesthesia. The resected specimen was 2 × 1 × 1 cm in size with a margin at the site of the probable tumor (Fig. 1E). Grossly, there was an approximate 5 mm depression in the skin. The operation took 23 min and there was minimal blood loss. The postoperative course was good, and the patient was discharged the day after surgery.

**Fig. 2:**
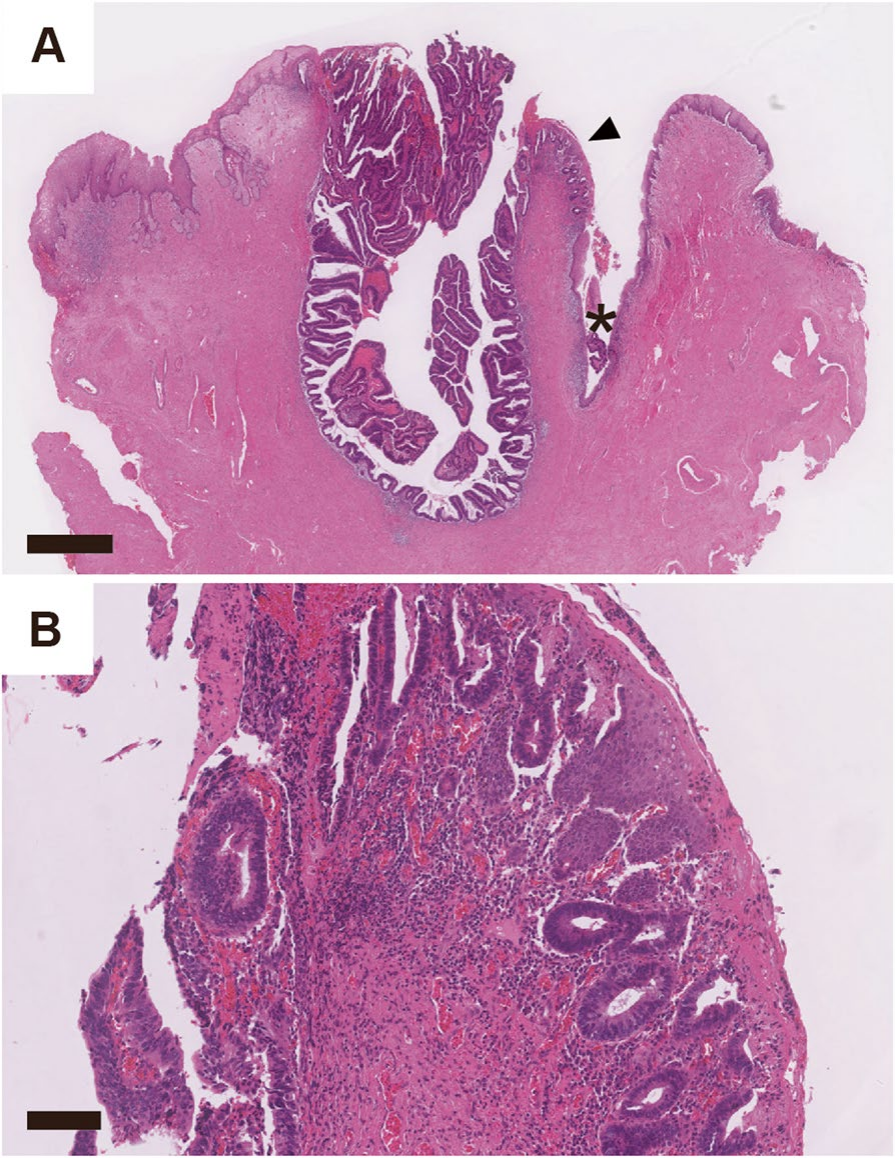
Pathological findings of hematoxylin–eosin staining. **A** Hematoxylin–eosin staining. A magnified view of the arrowhead is shown in (**B**). The asterisk indicates contamination. Magnification: 12.5 × , scale bar: 1000 µm. **B** Magnified view of the arrowhead in (**A**). Magnification: 100 × , scale bar: 100 µm

Histologic examination revealed that the tumor was situated in the mucosa covered by squamous epithelium, some portion of which had sebaceous glands. The tumor was a cystic lesion invaginating from the surface and was lined by a tubulovillous proliferation of dysplastic columnar epithelium (Fig. 2A). The nuclei were enlarged, and nucleoli were prominent. The mitotic activity was brisk. There were some fused glands, which were especially apparent in the tissue obtained in biopsy (Fig. 1A). Some dysplastic glands were on the mucosal surface and continuous with the non-neoplastic squamous epithelium (Fig. 2A, B). The tumor was well-circumscribed, with no infiltrative growth or lymphovascular infiltration. Sebaceous glands were the only non-dysplastic glandular elements present in the vicinity of the tumor; Skene glands, minor vestibular glands, endometriosis, ectopic intestinal mucosa, and intestinal metaplasia were not identified. A portion of Bartholin gland was in the periphery of the resected specimen, apart from the tumor.

**Fig. 3:**
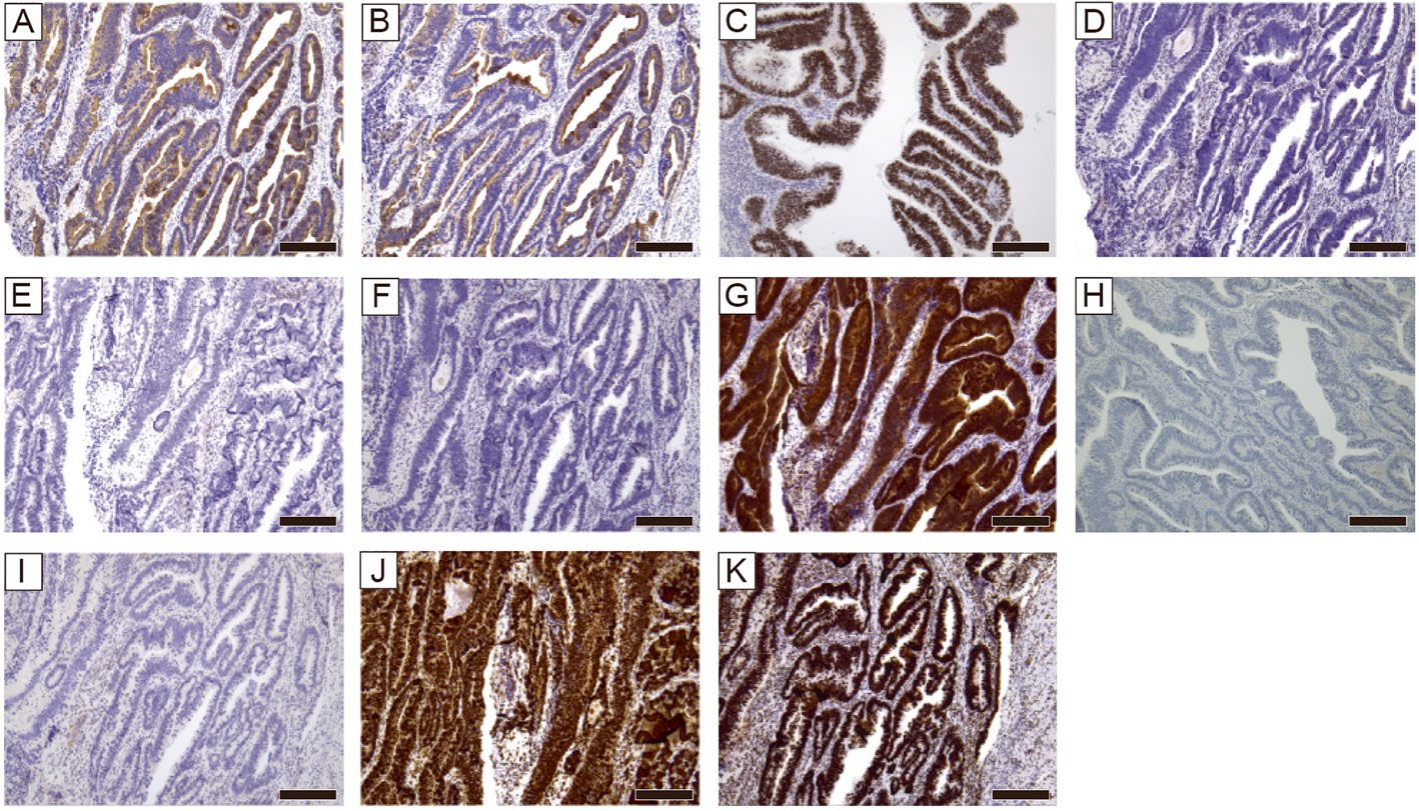
Pathological findings of immunohistochemistry. **A** Cytokeratin 20. Magnification: 100 × , scale bar: 200 µm. **B** Cytokeratin 7. Magnification: 100 × , scale bar: 200 µm. **C**. CDX2. Magnification: 100 × , scale bar: 200 µm. **D** Estrogen receptor. Magnification: 100 × , scale bar: 200 µm. **E** Progesterone receptor. Magnification: 100 × , scale bar: 200 µm. **F** PAX8. Magnification: 100 × , scale bar: 200 µm. **G** p16. Magnification: 100 × , scale bar: 200 µm. **H** HPV. Magnification: 100 × , scale bar: 200 µm. **I** p53. Magnification: 100 × , scale bar: 200 µm. **J** PMS2. Magnification: 100 × , scale bar: 200 µm. **K** MSH6. Magnification: 100 × , scale bar: 200 µm

IHC results were as follows: cytokeratin (CK) 20: positive, CK7: focal positive, CDX2: positive, estrogen receptor (ER): negative, progesterone receptor (PgR): negative, and PAX8: negative (Fig. 3A–F). p16 expression was strong and diffusely positive (Fig. 3G), and human papillomavirus (HPV) was negative (Fig. 3H). p53 was null pattern (Fig. 3I). PMS2 and MSH6 expressions were detected (Fig. 3J, K). On the basis of these results, the patient was diagnosed with primary adenocarcinoma of intestinal-type of the vulva. A gene panel test (FoundationOneCDx assay, Foundation Medicine, Inc., Cambridge, MA, USA) was performed on the tumor portion of the explanted specimen. TMB was high, at 13 mutations/megabase (Mut/Mb). The tumor was microsatellite stable. Gene mutations in *TP53*, *KEL*, *RB1*, *RNF43*, *PTEN*, *GNAS*, and *PIK3CA* were detected (Table 1).

**Table 1:** Gene mutations in the tumor specimen detected by FoundationOneCDx

Gene	Alteration	VAF (%)
*TP53*	splice site 672 + 1G > T	67.9
*KEL*	M1T	55.7
*RB1*	I815fs*11	40.6
*RNF43*	E258*	36.4
*PTEN*	T319fs*1	23.0
*GNAS*	R201H	1.9
*PIK3CA*	E453K	1.8

The patient did not receive any adjuvant therapy. At 30 months postoperation, there has been no recurrence.

## Discussion and conclusions

Thirty cases of adenocarcinoma of intestinal-type of the vulva or vagina have been reported. The cases were reported in a wide range of countries and races, and the mean patient age is 54.1 (31–69) years (Table 2) [3–30]. Symptoms of adenocarcinoma of intestinal-type of the vulva include pain, itching, and bleeding. In the early stage of cancer or precancerous lesion, as in the present case, the patient may present only with a mass (Table 2). One report suggests that adenocarcinoma of intestinal-type of the vulva may have a poor prognosis and that endoscopic follow-up of the colon is mandatory because of the high tendency to complicate gastrointestinal tumors [18]. In most cases, however, the clinical courses are gradual, and the prognosis is good (Table 2).

**Table 2:** Cases of intestinal-type adenocarcinoma of vulva and vagina

Year	First Author	Country	Race	Age	Symptom	Location	Maximum tumor size (cm)	Operation	Adjuvant therapy	Stage	LN meta	Follow (month)	Prognosis	CK20	CK7	CEA	CDX2	p16	ER	PR
1978	Tiltman	South Africa	NA	50	dyspareunia, discharge	external urethral meatus	2	RV + LND	none	II	positive	12	NED	NA	NA	-	NA	NA	NA	NA
1988	Fox	UK	NA	35	discharge, bleeding	left lateral and anterior viginal wall	5	WLE	none	NA	NA	NA	NA	NA	NA	NA	NA	NA	NA	NA
1991	Mortensen	Denmark	NA	43	discharge, bleeding	posterior rim of hymenal caruncle	1.7	WLE	none	NA	NA	27	rec 23 m: re-resection	NA	NA	+	NA	NA	NA	NA
1993	Kennedy	USA	NA	54	vulvar mass	left posterior vulva	2	RV + LND	none	I	negative	120	NED	+	+	-	NA	NA	NA	NA
1993	Kennedy	USA	NA	63	none	frenulum of labia minor	1.5	WLE	none	I	negative	48	NED	NA	NA	NA	NA	NA	NA	NA
1995	Ghamande	USA	NA	67	bleeding	frenulum of labia minor	1.7	RV + LND	none	I	negative	17	NED	NA	NA	+	NA	NA	NA	NA
1999	Nagar	UK	NA	36	bleeding	anterolateral upper vaginal wall	NA	WLE	NA	NA	NA	NA	NA	NA	NA	NA	NA	NA	NA	NA
1999	Willen	Sweden	NA	57	none	left posterior vestibule	1	WLE	none	I	negative	26	NED	NA	NA	+	NA	NA	NA	NA
2001	Mudhar	UK	NA	56	bleeding, discharge	posterior vaginal wall	1	WLE	none	I	negative	12	NED	+	-	+	NA	NA	NA	NA
2001	Zaidi	USA	African	43	vulvar mass, discomfort, bleeding	frenulum of labia minor	5	RV + LND	none	NA	negative	18	NED	NA	NA	+	NA	NA	-	-
2001	Rodriguez	Spain	NA	69	vulvar mass, itchy	right labia major	1.5	WLE	none	I	negative	36	NED	+	+	+	NA	NA	-	-
2003	Liu	Taiwan	Asian	49	NA	left labia major	2	RV + LND	none	I	negative	24	NED	NA	NA	NA	NA	NA	NA	NA
2004	Dube	Canada	NA	58	vulvar mass, discomfort, burning	right labia major	1.5	RV	none	I	negative	16	NED	+	+	NA	NA	NA	-	-
2005	Lee	Korea	Asian	61	spotting	vaginal introitus (11 o'clock)	2	WLE + laser	none	I	negative	9	rec 9 m: WLE	+	+	NA	NA	NA	NA	NA
2006	Tjalma	Belgium	NA	55	bleeding	posterior and anterior lower vaginal wall	4.5	PE	none	II	negative	20	NED	+	+	NA	NA	NA	-	-
2012	Cormio	Italy	NA	59	discomfort, burning	left posterior vestibule	NA	RV + LND	chemo	NA	NA	54	DOD	+	+	NA	NA	NA	NA	NA
2012	Cormio	Italy	NA	42	none	vulva	1	RV + LND	none	NA	NA	39	NED	+	+	NA	NA	NA	NA	NA
2012	Karkouche	France	Caucasian	31	none	bilateral labia minor	NA	WLE	none	NA	NA	15	NED	+	-	NA	NA	NA	NA	NA
2013	Wessel	Belgium	Caucasian	68	discharge, vulvar mass, pain	posterior vaginal introitus	1.3	WLE	none	NA	NA	25	NED	+	+	+	NA	NA	-	-
2013	Musella	Italy	NA	57	vulvar mass, bleeding	right labia major	5	RV + LND	neoadjuvant chemo (PTX + CDDP)	III	positive	4	NED	+	-	+	+	+	-	NA
2016	Sui	China	Asian	43	vulvar mass, itchy	vaginal introitus	1.5	WLE	chemo (PTX + CBDCA)	I	negative	24	NED	-	+	NA	NA	+	-	-
2016	Tepeoglu	Turkey	NA	40	vulvar discomfort, bleeding	left labia minor	2	WLE + LND	none	III	positive	38	NED	+	+	+	+	NA	NA	NA
2017	Matsuzaki	Japan	Asian	68	vulvar mass	vestibule	4	WLE	none	in situ	negative	60	NED	+	-	NA	+	NA	NA	NA
2017	Lee	Korea	Asian	64	vulvar mass, itchy	right labia major	4	WLE	none	II	negative	12	NED	+	+	+	+	+	NA	NA
2017	He	China	NA	63	vulvar mass	frenulum of labia minor	2	WLE	none	NA	NA	26	NED	+	+	+	+	-	-	-
2019	Kurita	Japan	Asian	63	bleeding	vestibule	2	WLE + LND	RT	I	negative	12	NED	+	-	NA	+	NA	NA	NA
2019	Kaltenecker	USA	African	53	vulvar mass, itchy, pain, discharge	bilateral labia minor	6	WLE	chemo (PTX + CBDCA) + RT	IV	positive	12	DOD	+	-	+	NA	NA	NA	NA
2019	Ugwu	Nigeria	African	40	vulvar mass, bleeding, pain	posterior lower vaginal wall	6	WLE	CCRT	I	negative	NA	NED	NA	NA	NA	NA	NA	NA	NA
2022	Moscoso	Spain	NA	66	itchy, discomfort	left labia minor	2	WLE + LND	none	I	negative	12	NED	+	-	+	+	+	-	-
2022	Sabri	USA	NA	62	bleeding, dysuria, cramping	right upper vaginal wall	3	none	CCRT	IV	NA	NA	NED	+	-	+	+	-	-	-
2022	present case	Japan	Asian	63	vulvar mass	posterior vulva (7 o'clock)	1	WLE	none	I	negative	27	NED	+	+	NA	+	+	-	-

*UK* United Kingdam, *USA* United States of America, *WLE* Wide local excision, *RV* Radical vulvectomy, *PE* Pelvic exenteration, *Chemo* Chemotherapy, *PTX* Paclitaxel, *CDDP* Cisplatin, *CBDCA* Carboplatin, *CCRT* Concurrent chemoradiotherapy, *RT* Radiation therapy, *LN* Lymph node, *NED* No evidence of disease, *DOD* Died of disease, *NA* Not available

Intestinal-type neoplasms of the vagina were reported to be like the adenoma-carcinoma sequence in colorectal tumors [31]. However, intestinal-type neoplasms of the vulva are rarer, and the adenoma-adenocarcinoma sequence has not been discussed. There are no reports of genetic analysis of intestinal-type adenomas of the vulva, and there have been only few reports of genetic analysis of adenocarcinoma of intestinal-type of the vulva or intestinal-type adenomas/adenocarcinomas of the vagina. In the current case, which was a as a precancerous lesion diagnosed as tubulovillous adenoma with high-grade dysplasia of the vulva, genetic analysis was performed and high TMB was detected, accompanied by many cancer-associated gene mutations. This lesion may be in the process of carcinogenesis, and the genetic mutations may be related to malignant transformation.

In the context of histopathological diagnosis considering tumor evolution, this case is very important. Adenocarcinoma of intestinal-type of the vulva resembles colorectal villous adenoma [32]. However, adenoma of intestinal-type of the vulva as a non-invasive, precancerous lesion has not been clearly defined. The tumor in the current case is an epithelial tumor with intestinal traits; following the classification of colorectal tumors, the most similar histomorphology is tubulovillous adenoma with high-grade dysplasia. Conventional colorectal adenoma recapitulates the normal crypt architecture of the mucosa in typical and early stage lesions, while some lesions also develop a villiform architecture. In the WHO classification, conventional colorectal adenoma is divided into tubular adenoma, tubulovillous adenoma, and villous adenoma depending on the ratio of tubular to villous structures [33]. Conventional colorectal adenoma is further divided into tumors with low-grade dysplasia and high-grade dysplasia [33]. Based on the context of colorectal adenoma, this case was diagnosed as tubulovillous adenoma with high-grade dysplasia. Fox et al. first reported a vaginal intestinal-type adenoma in 1988 [6]. Vitrey et al. reported low-grade adenoma of the vulva in 2003 [34]. In 2012, Karakouch et al. reported multiple tubulovillous adenomas of the vulva, including adenocarcinoma [19]. However, our report is the first case of tubulovillous adenoma with high-grade dysplasia that is expansile in the direction of the mucosa. We reviewed previous reports of adenocarcinoma of intestinal-type of the vulva, and the report by Willén et al. is likely a villous adenoma with an uncertain degree of dysplasia in the context of a colorectal adenoma [10]. Matsuzaki et al. reported villoglandular adenocarcinoma of the vulva without stromal invasion [24], which could also be considered tubulovillous adenoma with high-grade dysplasia. The cases of adenocarcinoma of intestinal-type of the vulva are considered to have a good prognosis, but it may be possible to redefine some of them as “adenoma.”

IHC plays an important role in diagnosis. It is necessary to exclude metastatic gastrointestinal cancers, such as metastatic colorectal cancer. Typically, normal intestinal epithelium is CK7 negative, CK20 positive, and CDX2 positive. Most colorectal cancers are CK7 negative, CK20 positive, and CDX2 positive, while rectal cancers tend to be CK7 positive [35]. The present case was also CK7-positive. In previous reports, among the 21 cases in which IHC for CK20 and CK7 were performed, 20 cases (95%) were positive for CK20 and 13 cases (62%) were positive for CK7 (Table 2). CDX2 is expressed in the mucosal epithelium from the duodenum to the rectum and is also positive in intestinal-type neoplasms of the lower female genital tract. In previous reports, IHC for CDX2 was positive in all eight cases in which it was performed (Table 2). ER and PgR were mostly negative, as observed in the present case (Table 2). Notably, p16 was positive in the present case. Five other cases had IHC findings for p16, four cases of which were positive (Table 2). In another report, reverse transcription polymerase chain reaction for HPV in p16-positive adenocarcinoma of intestinal-type of the vulva did not detect HPV type 16, but only low-risk HPV [29]. HPV was negative in the present case. The significance of HPV status is unclear and further study is needed. In the present case, p53 staining was null, which may reflect the *TP53* mutation described below.

The surgical technique for intestinal-type neoplasms of the vulva has not been established; however, complete resection with sufficient margin is presumed to be important. Although there is one report of a patient who received neoadjuvant chemotherapy followed by surgery [21], most cases are preceded by surgery (Table 2). The most common surgical techniques are wide local excision and radical vulvectomy, with lymph node dissection in some cases (Table 2). In the present case, the lesion was less than 2 cm grossly and there were no suspicious findings of lymph node or distant metastasis on imaging. Because the depth of invasion could not be accurately determined by biopsy, wide local excision was performed. There was a sufficient margin, and no adjuvant therapy was performed. For most other cases in which tumors were removed by surgery, adjuvant therapy was not used (Table 2).

Notably, a variety of cancer-associated mutations were detected in the current case. *TP53*, *KEL*, *RB1*, *RNF43*, and *PTEN* mutations showed high variant allele frequency and were considered clonal mutations. The variant allele frequencies for *PIK3CA* and *GNAS* mutations were very low and were considered to be subclonal mutations. In 2019, Shuangshoti et al. reported tubulovillous adenoma of the vagina with *KRAS* and *APC* mutations, despite the absence of high-grade dysplasia or adenocarcinoma [36]. This type of neoplasia may have accumulated cancer-associated genetic mutations in an early stage of evolution. In the gastrointestinal tract, except the colorectum, the histopathologic pattern of adenocarcinoma of intestinal-type is common in the esophagus [35], stomach [37], and duodenal papilla [38]. Outside of the gastrointestinal tract, adenocarcinoma of intestinal-type is most common in the sinuses and is the second most common histologic type of primary sinus cancer [39]. Rare cases of adenocarcinoma of intestinal-type of the tongue [40], gallbladder [41], lung [42], bladder [43], and ureter [44] as primary sites have been reported. In cancers with histopathology showing adenocarcinoma of intestinal-type, *TP53* and *KRAS* mutations are often detected. *TP53* mutations are particularly frequent in adenocarcinoma of intestinal-type of the stomach and primary sinus [39, 45]. Among adenocarcinomas of intestinal-type of the duodenal papilla, *TP53* and *KRAS* mutations were reported in approximately 40% of cases and *RNF43* mutations in approximately 15% of cases [38]. Another study reported mutations in *KRAS*, *PIK3CA,* and *SMAD4* [46]. In intraductal papillary mucinous neoplasms of the pancreas of the intestinal type, the frequency of *KRAS* mutations is approximately 50% [47]. There is also a report of adenocarcinoma of intestinal-type with *KRAS* mutation arising from a mature cystic teratoma of the ovary [48]. In contrast, *KRAS* and *BRAF* mutations are rare in adenocarcinomas of intestinal-type of the sinus [39].

The present case was a precancerous lesion and not treated with adjuvant therapy. However, if the patient was in an advanced stage or had a recurrence with malignant transformation, additional treatment options may have needed to be considered. Potentially actionable genetic mutations found in this case may help establish effective targeted therapy. Loss or activation mutations in *PTEN* activate the phosphoinositide 3-kinases (PI3K)/protein kinase B (AKT)/mammalian target of rapamycin (mTOR) pathway [49] and predict sensitivity to poly (ADP-ribose) polymerase (PARP) inhibitors [50]. *PIK3CA* mutation also activates the PI3K/AKT/mTOR pathway [49]. Therefore, depending on the course of the disease, the combination of mTOR inhibitors or PARP inhibitors may be considered. *GNAS* mutations activate ERK/MAPK signals [51]. In this case, a mitogen-activated protein kinase inhibitor may have been useful.

In the present case, TMB was high, at 13 Mut/Mb. TMB is an indicator of response to immunotherapy [52]. The KEYNOTE-158 study showed that advanced solid tumors with TMB ≥ 10 were more likely to respond to pembrolizumab, an anti-PD-1 antibody [53]. If the patient had been in an advanced stage or had a recurrence with malignant transformation, an immune checkpoint inhibitor like pembrolizumab may have been a treatment option. Therefore, in intestinal-type neoplasms of the vulva, a gene panel test may be helpful to select treatment options. In addition, while present case was microsatellite stable, some reports have shown that microsatellite instability–high and mismatch repair deficiency is more frequent in gastric adenocarcinoma of intestinal-type [54, 55], and microsatellite status and IHC for mismatch repair protein may also be checked in intestinal-type neoplasms of the vulva.

In conclusion, here we report a case of tubulovillous adenoma with high-grade dysplasia of the vulva harboring multiple cancer-associated mutations and high TMB. In cases of intestinal-type neoplasms of the vulva, it may be helpful to check TMB and gene mutations for treatment selection.

## Data Availability

All data generated or used during the study are available from the corresponding author by request.

## Abbreviations

IHC: Immunohistochemistry
TMB: Tumor mutational burden
HE: Hematoxylin–eosin
CK: Cytokeratin
ER: Estrogen receptor
PgR: Progesterone receptor
HPV: Human papillomavirus
PI3K: Phosphoinositide 3-kinases
AKT: Protein kinase B
mTOR: Mammalian target of rapamycin
PARP: Poly (ADP-ribose) polymerase
Mut/Mb: Mutations/megabase
MSI: Microsatellite instability
